# Performance of ChatGPT on Nursing Licensure Examinations in the United States and China: Cross-Sectional Study

**DOI:** 10.2196/52746

**Published:** 2024-10-03

**Authors:** Zelin Wu, Wenyi Gan, Zhaowen Xue, Zhengxin Ni, Xiaofei Zheng, Yiyi Zhang

**Affiliations:** 1 Department of Bone and Joint Surgery and Sports Medicine Center, The First Affiliated Hospital of Jinan University, No.613, Huangpu Avenue West, Tianhe District, Guangzhou, Guangdong, 510630, China, 86 18002255355; 2 Department of Joint Surgery and Sports Medicine, Zhuhai People’s Hospital, Zhuhai City, China; 3 School of Nursing, Yangzhou University, Yangzhou, China

**Keywords:** artificial intelligence, ChatGPT, nursing licensure examination, nursing, LLMs, large language models, nursing education, AI, nursing student, large language model, licensing, observation, observational study, China, USA, United States of America, auxiliary tool, accuracy rate, theoretical

## Abstract

**Background:**

The creation of large language models (LLMs) such as ChatGPT is an important step in the development of artificial intelligence, which shows great potential in medical education due to its powerful language understanding and generative capabilities. The purpose of this study was to quantitatively evaluate and comprehensively analyze ChatGPT’s performance in handling questions for the National Nursing Licensure Examination (NNLE) in China and the United States, including the National Council Licensure Examination for Registered Nurses (NCLEX-RN) and the NNLE.

**Objective:**

This study aims to examine how well LLMs respond to the NCLEX-RN and the NNLE multiple-choice questions (MCQs) in various language inputs. To evaluate whether LLMs can be used as multilingual learning assistance for nursing, and to assess whether they possess a repository of professional knowledge applicable to clinical nursing practice.

**Methods:**

First, we compiled 150 NCLEX-RN Practical MCQs, 240 NNLE Theoretical MCQs, and 240 NNLE Practical MCQs. Then, the translation function of ChatGPT 3.5 was used to translate NCLEX-RN questions from English to Chinese and NNLE questions from Chinese to English. Finally, the original version and the translated version of the MCQs were inputted into ChatGPT 4.0, ChatGPT 3.5, and Google Bard. Different LLMs were compared according to the accuracy rate, and the differences between different language inputs were compared.

**Results:**

The accuracy rates of ChatGPT 4.0 for NCLEX-RN practical questions and Chinese-translated NCLEX-RN practical questions were 88.7% (133/150) and 79.3% (119/150), respectively. Despite the statistical significance of the difference (*P*=.03), the correct rate was generally satisfactory. Around 71.9% (169/235) of NNLE Theoretical MCQs and 69.1% (161/233) of NNLE Practical MCQs were correctly answered by ChatGPT 4.0. The accuracy of ChatGPT 4.0 in processing NNLE Theoretical MCQs and NNLE Practical MCQs translated into English was 71.5% (168/235; *P*=.92) and 67.8% (158/233; *P*=.77), respectively, and there was no statistically significant difference between the results of text input in different languages. ChatGPT 3.5 (NCLEX-RN *P*=.003, NNLE Theoretical *P*<.001, NNLE Practical *P*=.12) and Google Bard (NCLEX-RN *P*<.001, NNLE Theoretical *P*<.001, NNLE Practical *P*<.001) had lower accuracy rates for nursing-related MCQs than ChatGPT 4.0 in English input. English accuracy was higher when compared with ChatGPT 3.5’s Chinese input, and the difference was statistically significant (NCLEX-RN *P*=.02, NNLE Practical *P*=.02). Whether submitted in Chinese or English, the MCQs from the NCLEX-RN and NNLE demonstrated that ChatGPT 4.0 had the highest number of unique correct responses and the lowest number of unique incorrect responses among the 3 LLMs.

**Conclusions:**

This study, focusing on 618 nursing MCQs including NCLEX-RN and NNLE exams, found that ChatGPT 4.0 outperformed ChatGPT 3.5 and Google Bard in accuracy. It excelled in processing English and Chinese inputs, underscoring its potential as a valuable tool in nursing education and clinical decision-making.

## Introduction

The large language model (LLM) technology is a stepping stone in the evolution of artificial intelligence (AI) [1,2]. Through the analysis of a large database, the primary module generates a logical and plain text response to the user’s query promptly following the user’s textual input [3]. Currently, popular AI software includes ChatGPT 4.0, ChatGPT 3.5, and Google Bard, and research indicates that these 3 AI algorithms perform well when answering queries about lung cancer [4]. AI tools are the result of the advancement of science and technology, and the advent of revolutionary tools will alter the way people learn and work, which is an irreversible trend.

ChatGPT has been controversial since its public release in November 2022 due to its powerful text generation capabilities, and attention has been focused on students using ChatGPT for essay writing and assignment plagiarism [5-7]. With the birth of regulatory software such as GPTZero, AI-Text-Classifier, and ChatGPT Detector, people gradually focused on the application of ChatGPT, trying to explore and expand the application field of ChatGPT. The study found that ChatGPT showed both professionalism and empathy in answering general public health questions [8]. ChatGPT not only showed strong expertise in answering basic research directions but also followed evidence-based clinical decision-making [9,10]. Nevertheless, there may be some ethical problems in clinical application, and it is necessary to consider whether the use of ChatGPT will violate the rights and interests of patients [11-13]. Therefore, more and more researchers have placed the application field of ChatGPT in education [14]. The studies found that ChatGPT performed well on multiple-choice questions (MCQs) about otolaryngology and gynecology [15,16]. In addition, ChatGPT software can pass the Plastic Surgery Inservice Training Examination [17], the American Heart Association Basic Life Support Examinations [18], and the Taiwanese Pharmacist Licensing Examination [19]. ChatGPT is also able to solve higher-order problems related to medical biochemistry while also achieving satisfactory performance in surgical education and training [20,21]. However, ChatGPT is not a training tool for all exams, with the exception of the American Heart Association’s Advanced Cardiovascular Life Support (ACLS) exams and Taiwan’s Family Medicine Board Exam [18,22]. This might suggest that ChatGPT’s application areas may be limited by language and region in addition to speciality.

Both the United States and China have instituted licensing exams to regulate the qualifications of registered nurses [23]. China uses the National Nursing Licensure Examination (NNLE) [23], whereas the United States uses the National Council Licensure Examination for Registered Nurses (NCLEX-RN) [24], both of which seek to standardize the theoretical and practical foundations of nurses through standardized assessment procedures to ensure the professionalism of nurses who are entering the medical field. The content of nursing studies is not medically specialized but rather interdisciplinary and multidisciplinary [25]. On the basis of their nursing work, nurses are frequently required to comprehend clinical decisions made by physicians. As a result, it is easy for society to disregard the difficulty of nursing education and training, that is, the necessity of a medical foundation for the development of nursing expertise [26]. Presently, there are no professional nursing learning aids to assist nurses in gaining a better understanding of the professional medical issues encountered during the clinical learning process. Huge and intricate, the medical knowledge system necessitates repeated learning, even for specialists, in order to master specialized knowledge [27]. Despite the fact that many researchers attempt to implement various review strategies to increase the passage rate of nursing professional examinations, it is frequently difficult to popularize a single review strategy due to varying local practical policies [28]. No single revision method is appropriate for all individuals. How to assist nurses in gaining a deeper understanding of medical knowledge, enhancing their stockpile of professional theoretical knowledge, and increasing their exam pass rate is a pressing issue for nurses today.

The design of this research is cross-sectional. By incorporating NCLEX-RN and NNLE questions, we evaluated the precision of responses from ChatGPT 4.0, ChatGPT 3.5, and Google Bard. Concurrently, the translation feature of ChatGPT 3.5 was used to convert between Chinese and English, while an examination was conducted into the disparity in the rate of accurate responses provided by ChatGPT across various languages. The aim of this study is to offer a conceptual framework that supports the implementation of ChatGPT and advances nursing education and clinical application.

## Methods

### Design

With reference to Zong et al [29], we designed a cross-sectional study. The experimental data from our study had been recorded in an Excel file and uploaded as Multimedia Appendix 1. The STROBE Initiative [30] was used in this study and the STROBE Initiative checklist is available in Multimedia Appendix 2.

### Ethical Considerations

As this study does not involve interventional experiments on humans or animals, the research does not require approval per the Ethics Committee of the First Affiliated Hospital of Jinan University guidelines.

### Data Source

NCLEX-RN practice questions were compiled at the website “nurseslabs” [31]. There were no set questions on the official NCLEX-RN test; instead, a computer produced new questions with a minimum of 75 and a maximum of 265 depending on how accurate the preceding questions were. Thus, we got the most recent 2 sets of practice questions for the NCLEX-RN exam from the internet. In 2 practice sets, we compiled a total of 150 MCQs.

The NNLE question categories were divided into 2 sections: nursing theory and nursing practice, each containing 120 MCQs. On the website “baidu” [32], we used the most current 480 NNLE-MCQs from the 2022 and 2021 exams that were accessible. According to the classification of nursing theory examination and nursing practice, the questions for 2022 and 2021 were merged and then separated into NNLE Theoretical MCQs (n=240) and NNLE Practical MCQs (n=240).

### Procedures

**Figure 1.**
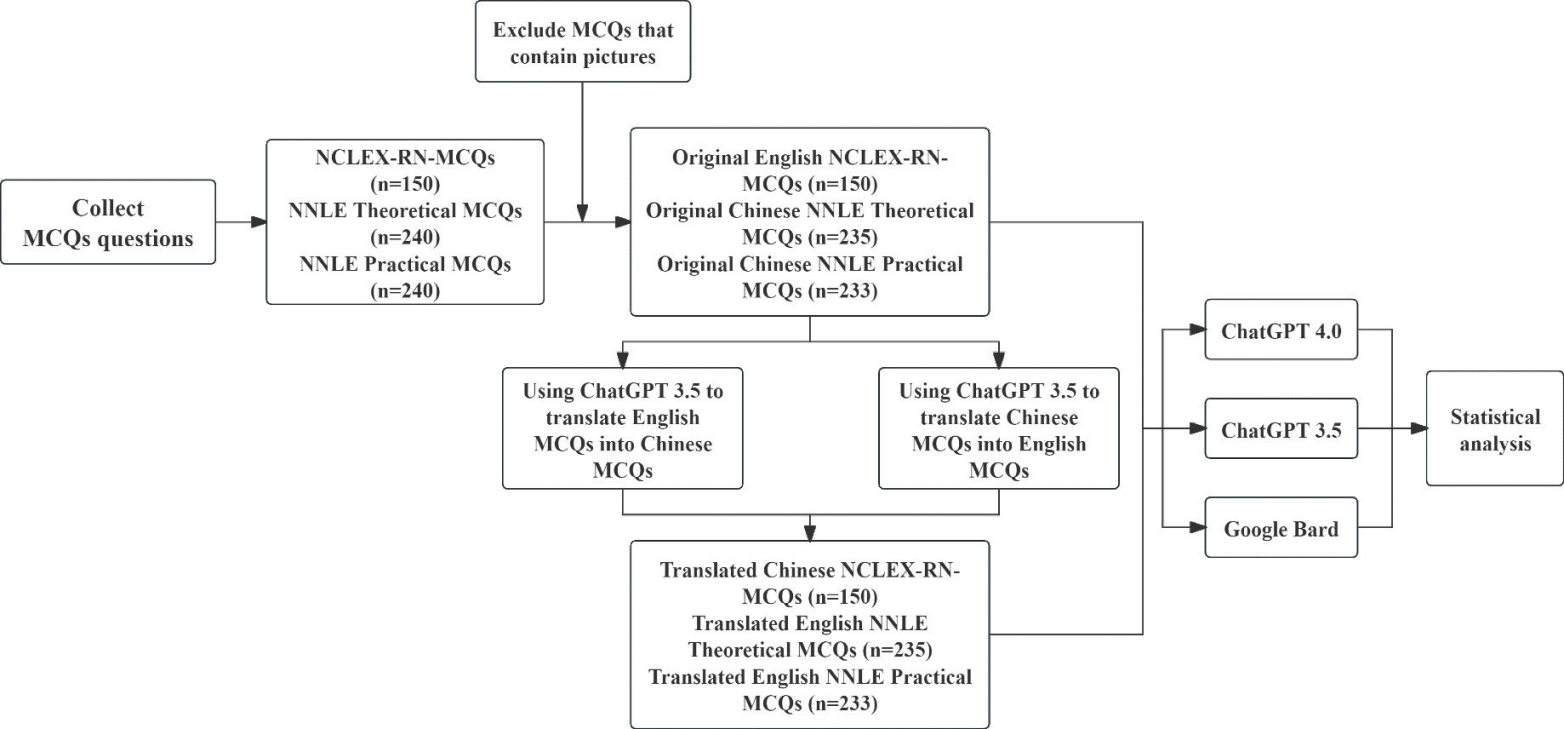
Diagrammatic representation of the progression of exploratory application experiments. MCQ: multiple-choice question; NCLEX-RN: National Council Licensure Examination for Registered Nurses; NNLE: National Nursing Licensure Examination.

According to the research stages (Figure 1), we translated the original English NCLEX-RN-MCQs into the Chinese version of the NCLEX-RN-MCQs. The original NNLE queries were written in Chinese, and we also translated them into English. To avoid systematic errors induced by differences in translation quality during the translation process, ChatGPT 3.5 was used to translate both from Chinese to English and from English to Chinese. We checked the language both before and after translating using ChatGPT 3.5 to translate between Chinese and English, as well as English and Chinese. About some clear translation mistakes, we entered the incorrect translation points in ChatGPT 3.5’s dialog box and requested that ChatGPT 3.5 retranslate the text.

**Figure 2.**
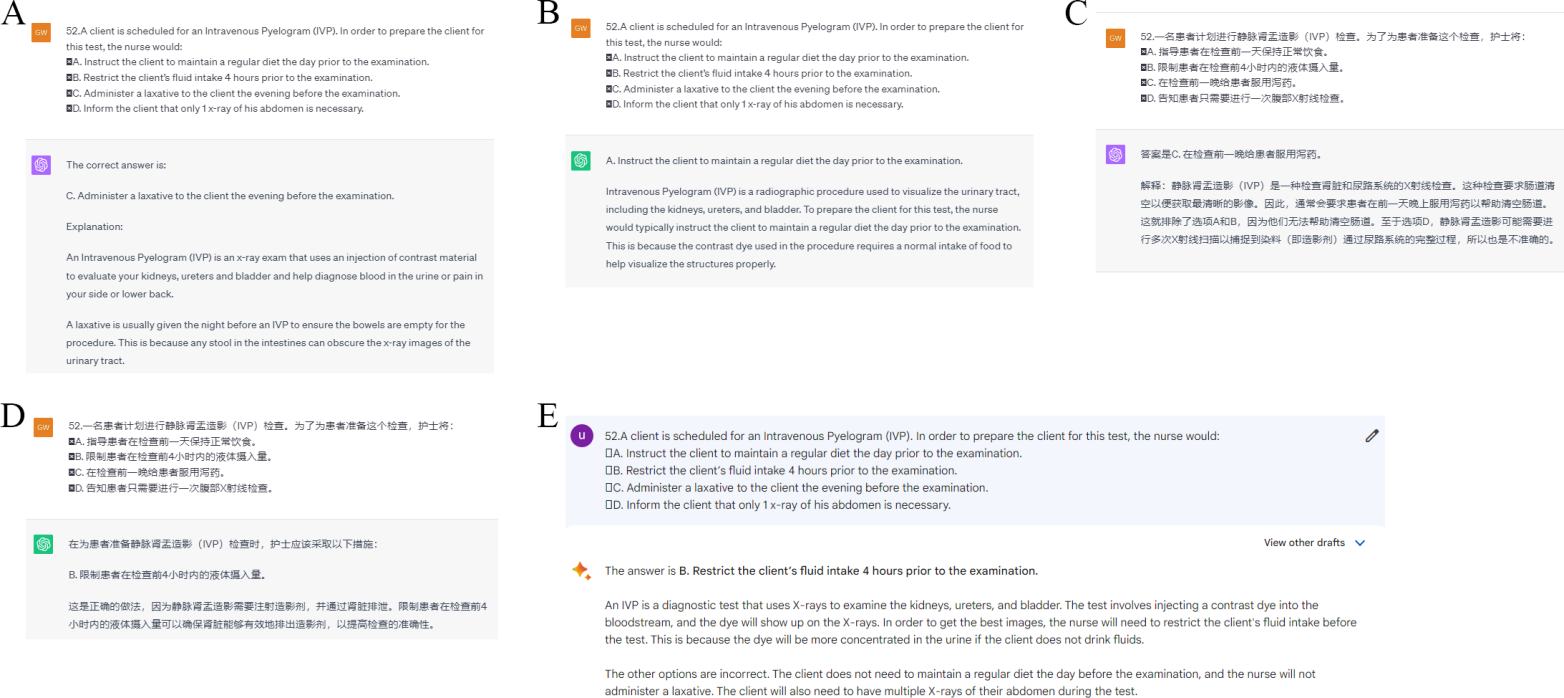
(A) English multiple-choice questions (MCQs) input in ChatGPT 4.0. (B) English MCQs input in ChatGPT 3.5. (C) Chinese MCQs input in ChatGPT 4.0. (D) Chinese MCQs input in ChatGPT 3.5. (E) English MCQs input in Google Bard.

Entered all questions on ChatGPT 4.0 (Figure 2Aand C) [33] and ChatGPT 3.5 (Figure 2B and D) [34] as well as Google Bard (Figure 2E) [35], then recorded the responses. Both ChatGPT 4.0 and ChatGPT 3.5 support text input in non-English languages, whereas Google Bard only supports text input in English at this time. The use of “New chat” for each inquiry ensured the independence of each response because it prevented the AI from using context from previous interactions, thereby eliminating any learning or bias that may have been carried over from earlier questions. Additionally, no plugins were used with ChatGPT, and the “Chat history & training” option was deactivated to preserve the objectivity of each response.

### Data Analysis

SPSS program (version 26.0; IBM Corp) was used for statistical analysis. With reference to Zong et al [29]. Collected the responses from ChatGPT 4.0, ChatGPT 3.5, and Google Bard and converted them to the binary variables “true” or “false.” Pearson The *χ*^*2*^ test was used to compare the differences between various LLM software or the same software input in various languages. A difference was considered statistically significant when the *P* value was less than .05. Used the web-based VENN diagram drawing website “bioinfogp” [36] to draw VENN diagrams to display different AI software’s results for the same type of subject with various linguistic inputs. Last, bar charts were constructed from a portion of the data using GraphPad Prism 8.

## Results

### Overview

We collected 150 NCLEX-RN-MCQs in total. We excluded the image questions from the compiled NNLE-MCQs because the picture analysis of ChatGPT and Google Bard required the use of external plug-ins. After eliminating the image questions, there were a total of 235 NNLE Theoretical MCQs and 233 NNLE Practical MCQs left. Then, ChatGPT 3.5 converted NCLEX-RN-MCQs for English questions into the Chinese version and NNLE-MCQs into the English version.

### Performance of LLMs in Responding to English NCLEX-RN MCQs

**Figure 3.**
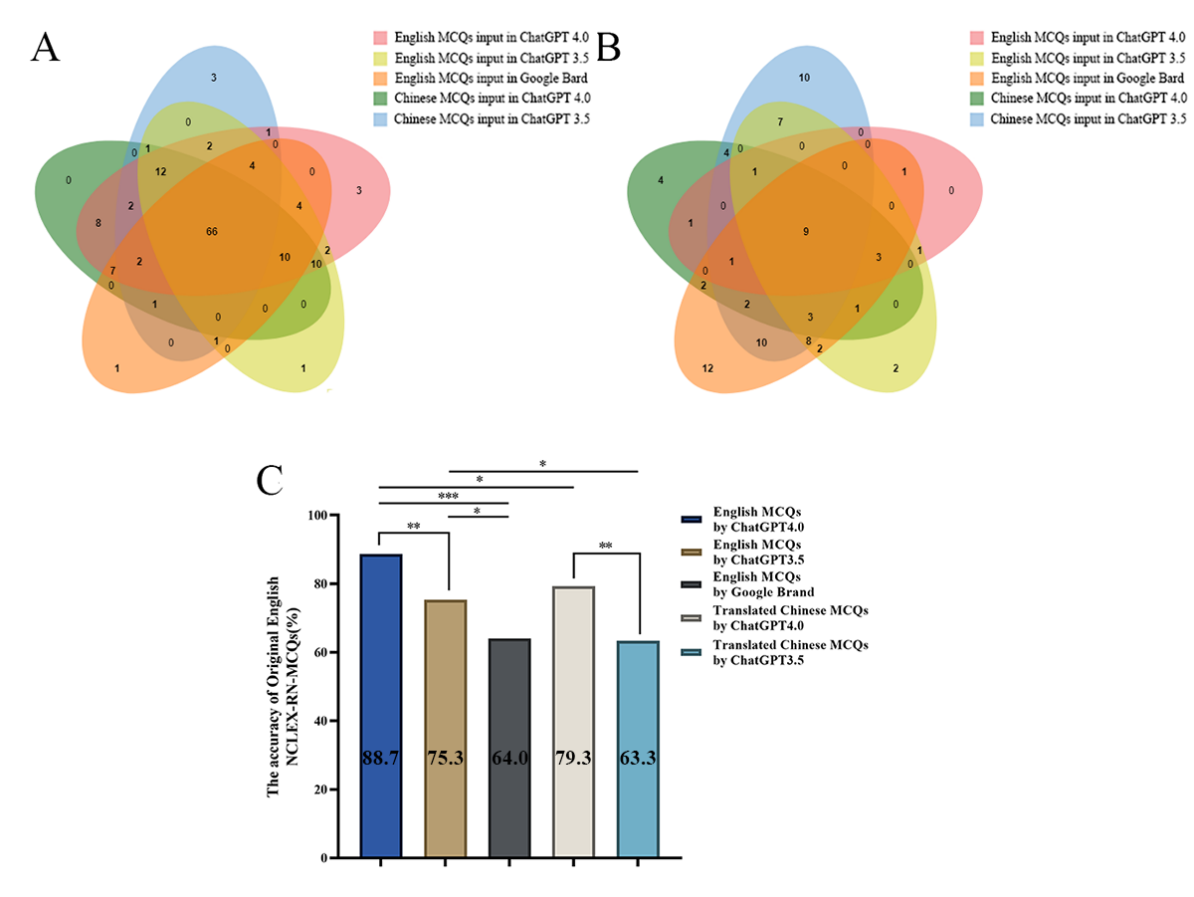
(A,B) VENN diagram shows the correct and incorrect intersection of NCLEX-RN practical questions in different large language models. (C) The correct rate of NCLEX-RN practical questions in various large language models. MCQ: multiple-choice question; NCLEX-RN: National Council Licensure Examination for Registered Nurses.

ChatGPT 4.0 had an accuracy rate of 88.67% (133/150) when answering NCLEX-RN MCQs in English, which was higher than ChatGPT 3.5 (113/150, 75.3%) and Google Bard (96/150, 64%) (Figure 3C). Statistically, ChatGPT 4.0 performed significantly better than the other 2 categories (ChatGPT 4.0 vs ChatGPT 3.5, *P*=.003; ChatGPT 4.0 vs Google Bard, *P*<.001) (Figure 3C). ChatGPT 3.5 was more accurate than Google Bard and the difference was statistically significant (*P*=.03) (Figure 3C).

### Performance of LLMs in Responding to Chinese NNLE-MCQs

**Figure 4.**
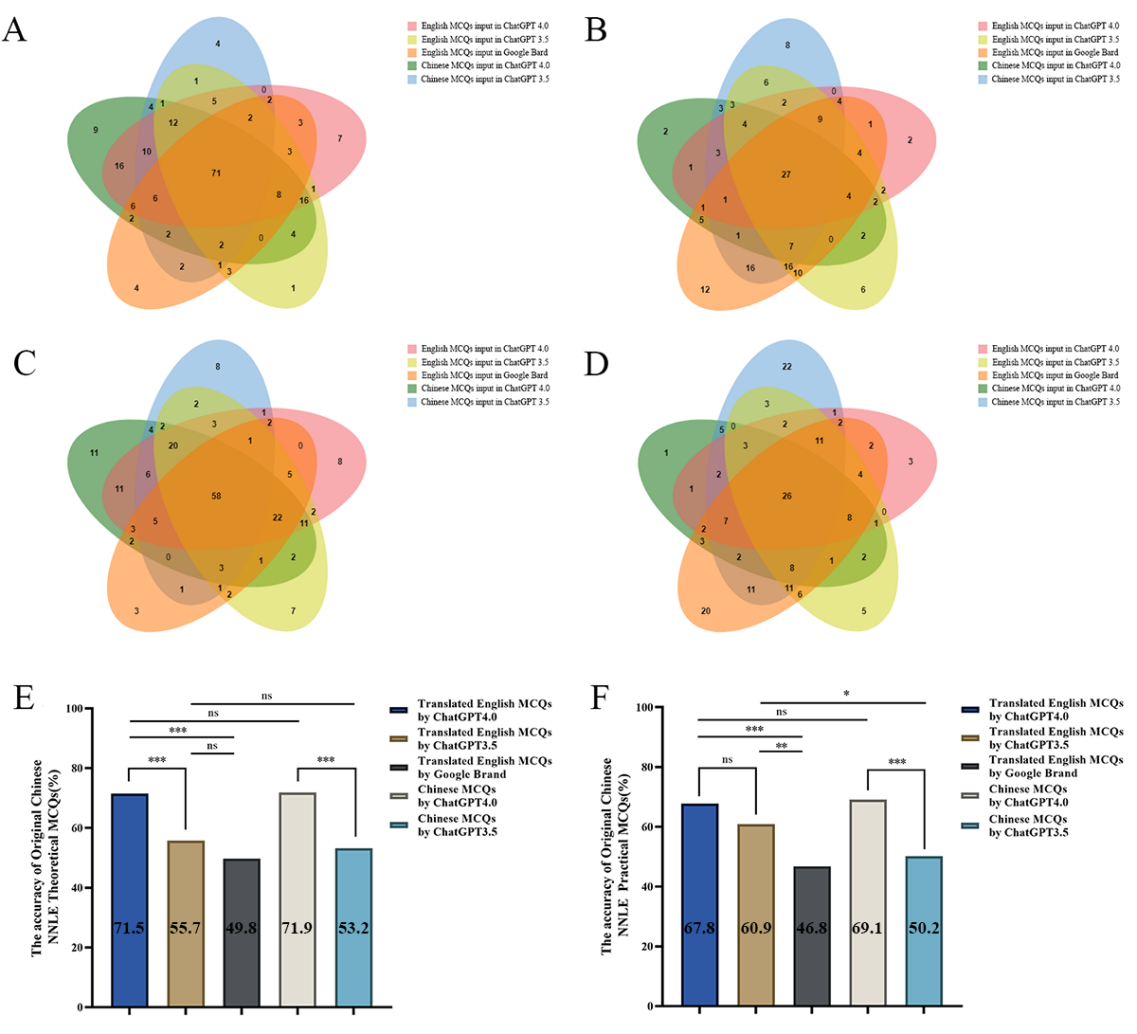
(A,B) VENN diagram shows the correct and incorrect intersection of NNLE theoretical MCQs in different large language models (LLMs). (C,D) VENN diagram shows the correct and incorrect intersection of NNLE practical MCQs in different LLMs. (E) The correct rate of NNLE theoretical MCQs in various LLMs. (F) The correct rate of NNLE practical MCQs in various LLMs. MCQ: multiple-choice question; NNLE: National Nursing Licensure Examination.

The difference between the correct rates of ChatGPT 4.0 and ChatGPT 3.5 in answering the Chinese version of NNLE theoretical MCQs (*P*<.001) and NNLE practical MCQs (*P*<.001) was statistically significant (Figure 4E and F). The correct rates of ChatGPT 4.0 answering NNLE theoretical MCQs and NNLE practical MCQs were 71.9% (169/235) and 69.1% (161/233), respectively, compared with 53.2% (125/235) and 50.2% (117/233) for ChatGPT 3.5 (Figure 4E and F).

### Performance and Variations of MCQs Input Into LLMs in Various Languages

After entering the Chinese-translated version of NCLEX-RN-MCQs into ChatGPT 4.0 and ChatGPT 3.5, we discovered that the accuracy rates were 79.3% (119/150) and 63.3% (95/150), respectively, with a statistically significant difference between the two (*P*=.002) (Figure 3C).

Then, we fed the English-translated version of NNLE Theoretical MCQs into ChatGPT 4.0, ChatGPT 3.5, and Google Bard and determined that their respective accuracy rates were 71.5 % (168/235), 55.7% (131/235), and 49.8% (117/235) (Figure 4E). ChatGPT 4.0 had a higher accuracy rate than ChatGPT 3.5 (*P*<.001) and Google Bard (*P*<.001) for the English-translated version of NNLE Theoretical MCQs while the difference was statistically significant (Figure 4E). ChatGPT 3.5 had a higher accuracy rate than Google Bard, but the difference was not statistically significant (*P*=.20) (Figure 4E).

The accuracy rates of ChatGPT 4.0, ChatGPT 3.5, and Google Bard were 67.8% (158/233), 60.9% (142/233), and 46.8% (109/233), respectively, when the English-translated version of NNLE Practical MCQs was inputted (Figure 4F). In terms of the English-translated version of NNLE Practical MCQs, the accuracy rates of both ChatGPT 4.0 (*P*<.001) and ChatGPT 3.5 (*P*=.002) were higher than those of Google Bard, and the difference was statistically significant; however, unlike before, the difference in accuracy rates between ChatGPT 4.0 and ChatGPT 3.5 was not statistically significant (*P*=.12) (Figure 4F).

When processing NCLEX-RN-MCQs, the accuracy of inputs in the original English version was statistically significantly higher than that of inputs translated into Chinese for both ChatGPT 4.0 (*P*=.03) and ChatGPT 3.5 (*P*=.02) (Figure 3C). The difference was not statistically significant between the accuracy of inputs in the original Chinese version and the inputs of the translated English version for both ChatGPT 4.0 (*P*=.92) and ChatGPT 3.5 (*P*=.58) when processing NNLE Theoretical MCQs (Figure 4E). The accuracy of ChatGPT 4.0’s inputs in the original Chinese version was higher than that of inputs translated into English when processing NNLE Practical MCQs, but this difference was not statistically significant (*P*=.77) (Figure 4F). Surprisingly, the accuracy of ChatGPT 3.5’s inputs in the original Chinese version was lower than that of inputs translated into English while dealing with NNLE Practical MCQs, and this difference was statistically significant (*P*=.02) (Figure 4F).

Figure 3A and B depicts, respectively, the intersection of correct and incorrect questions when NCLEX-RN practical questions were inputted into various LLMs in various languages. Similarly, Figure 4A and B depicts NNLE Theoretical MCQs, while Figure 4C and D depicts NNLE Practical MCQs. When the same questions were input into ChatGPT 4.0, ChatGPT 3.5, and Google Bard in English, ChatGPT 4.0 had the highest number (n for NCLEX-RN MCQs=14; n for NNLE Theoretical MCQs=33; n for NNLE Practical MCQs=26) of uniquely correct answers and the lowest number (n for NCLEX-RN MCQs=2; n for NNLE Theoretical MCQs=6; n for NNLE Practical MCQs=7) of uniquely incorrect answers among the 3 engines. Instead, Google Bard had a lower number (n for NCLEX-RN MCQs=2; n for NNLE Theoretical MCQs=10; n for NNLE Practical MCQs=6) of uniquely correct answers than ChatGPT 4.0 and the highest number (n for NCLEX-RN MCQs=26; n for NNLE Theoretical MCQs=34; n for NNLE Practical MCQs=36) of uniquely incorrect answers among the 3 engines when the MCQs were input into 3 engines in English. Likewise, after the questions were submitted in Chinese, we found that ChatGPT 4.0 (n for NCLEX-RN MCQs=35; n for NNLE Theoretical MCQs=61; n for NNLE Practical MCQs=63) gives more uniquely accurate responses than ChatGPT 3.5(n for NCLEX-RN MCQs=11; n for NNLE Theoretical MCQs=17; n for NNLE Practical MCQs=19) does.

## Discussion

### Principal Findings

This study is a cross-sectional study that collected a total of 618 nursing-related MCQs, including 150 NCLEX-RN practice questions and 468 NNLE actual exam questions. To observe differences between inputs in different languages, ChatGPT 3.5 was used exclusively for Chinese-to-English and English-to-Chinese translations. The results revealed that ChatGPT 4.0 had a significantly higher accuracy rate when handling English input for NCLEX-RN practical MCQs compared with ChatGPT 3.5 and Google Bard. Similarly, ChatGPT 4.0 also outperformed ChatGPT 3.5 in accuracy when processing the Chinese input of NNLE exam MCQs. Therefore, ChatGPT 4.0 has the potential to be an effective learning assistance software for ChatGPT users, and due to its powerful real-time text generation capabilities, it can also provide additional sources of information and reference for nursing decisions in clinical nursing work.

Despite being a tool that accepts input in different languages, ChatGPT has linguistic bias while processing text input, as this research has shown. ChatGPT 3.5 translates NCLEX-RN practical MCQs from English to Chinese. Following input, it was discovered that while interacting with English, ChatGPT 4.0 and ChatGPT 3.5 had accuracy rates that were noticeably greater than Chinese. When NNLE MCQs were input into ChatGPT in English, ChatGPT 4.0’s accuracy of the response was only somewhat less accurate than the Chinese input, while ChatGPT 3.5’s English input was even more accurate than the Chinese input. Although there may be some linguistic distortion when translating between languages using software, the findings of our cross-sectional investigation indicated that ChatGPT processes English input more accurately than Chinese input. I asked ChatGPT, an AI program that facilitates real-time communication, questions in an attempt to comprehend the logic behind handling input in various languages. In response, ChatGPT said that it can assess and respond to queries in several languages depending on the language of input. This capability stems from its training of various input kinds in various languages. As a result, the current discrepancy in accuracy caused by input in Chinese and English may be the result of ChatGPT receiving different amounts of training in different languages. This discrepancy may disappear with an increase in language training once ChatGPT becomes more well-known worldwide.

The low passage rate of nursing examinations is partly attributed to the lack of fundamental theoretical and clinical knowledge among nursing staff [24,37]. Researchers have tried to reform and innovate nursing education models within certain limits to improve knowledge levels and exam pass rates [28]. However, due to differences in language and local policies, it is challenging to widely implement a single educational model. MCQs are an effective method to assess student knowledge [38], but existing learning resources often require students to conduct independent searches to expand knowledge, adding to learning pressure and affecting the coherence of the learning process. ChatGPT’s big data analysis and rapid text feedback can help students consolidate and expand knowledge points while completing MCQ exercises [39]. Besides, ChatGPT 4.0 not only enhances the efficiency of nursing education [40] but also provides clinicians and nurses with objective information support based on evidence-based medicine and big data analysis in complex clinical scenarios [41]. For instance, the research discovered that ChatGPT 4.0 not only analyzed imaging data with acceptable accuracy and sensitivity but also assisted physicians in thinking outside the box and offering several helpful recommendations when making individualized clinical treatment choices for tumor patients [41]. Furthermore, ChatGPT may provide nurses with a customized and immersive learning experience, bolster their competence and self-assurance in overseeing remote patient care, and furnish them with the necessary abilities for remote patient monitoring, all of which can contribute to the enhancement of patient outcomes and care quality [42]. Additionally, ChatGPT may assist doctors in streamlining patient data organization and easing the burden of interpreting medical records in order to improve patient communication while doing therapeutic procedures [43].

According to this study and previous research findings, ChatGPT 4.0 is currently the most accurate and repeatable AI software among many LLMs. In answering questions related to electrocardiogram images [44], the Multi-Specialty Recruitment Assessment exam [45], dental professional issues [46], and analyzing radiology data [47], ChatGPT 4.0 provides more accurate and comprehensive responses compared with ChatGPT 3.5 and Google Bard. Since ChatGPT 4.0 is currently the only paid AI software compared with free-to-use LLMs like ChatGPT 3.5, Google Bard, and Bing, it is essential to compare its functionality with these free LLMs when exploring its real-world application value. The economic cost of use is also a factor that must be considered in the popularization and promotion of its application [48].

Assessing ChatGPT’s clinical application value in a manner that aligns with the training of experienced clinical workers is the same approach; upon passing the theory test, candidates will be deemed to possess fundamental medical theoretical knowledge and be capable of managing simple clinical scenarios [49]. The intricacy of clinical issues will then continuously increase as a result of ongoing training that corrects incorrect theoretical knowledge and clinical reasoning. Last, they get training to become highly repeatable and capable self-correcting clinical practitioners. ChatGPT has shown that it has a theoretical foundation for supporting clinical practice with its outstanding success in the qualifying exams of many clinical professions [15-22,45,46,49]. However, whether it is used as an auxiliary tool for self-learning and education, to support patient communication, or to aid in the analysis of complicated clinical circumstances, a commensurate regulatory system must be developed. In order to limit the circumstances in which ChatGPT is used, schools, hospitals, and publishing companies must first create pertinent policies [50]. Some examples of these policies include forbidding the use of ChatGPT during exams [51] and obtaining patient consent before using ChatGPT as an auxiliary tool in real clinical settings [52]. Authors must state that ChatGPT was not directly engaged in the creation of the text for the paper and are forbidden from claiming ChatGPT as an independent author [53]. Furthermore, the most immediate regulators of ChatGPT are its users. ChatGPT can assist with data collection and content integration, but the user has to take part in the quality review process of the content that ChatGPT generates, identify any problems in the responses that ChatGPT generates, and finish training ChatGPT via error correction and continuous input and output. Although many companies developing LLMs claim to avoid the collection and leakage of private information, as users of these software, it is also essential to ensure the content and quality of the input information. Users should intentionally avoid and delete personal and private information, thereby enhancing their personal oversight function during the use of the software. It is also crucial to seek the informed permission of other participants and make suitable declarations while using ChatGPT in public to prevent unwanted confrontations between doctors and patients, moral and ethical disagreements, and concerns with writing integrity.

### Implication

Our study has demonstrated that ChatGPT 4.0 exhibits a satisfactory accuracy rate in handling MCQs for the NCLEX-RN and NNLE exams, outperforming 2 other AI engines, ChatGPT 3.5 and Google Bard. Although there were differences in accuracy rates when the same questions were inputted in different languages, the overall accuracy of ChatGPT 4.0 remains commendable. Combined with conclusions from previous research, it can be inferred that ChatGPT 4.0 possesses the knowledge reserve necessary for application in medical education, learning, and clinical scenarios, with the potential to assist in managing complex clinical situations. To promote the rational application of ChatGPT 4.0 in the medical field, it is imperative for relevant authorities to develop effective and reasonable regulatory mechanisms and supervisory bodies in the future. This will ensure that ChatGPT 4.0, a powerful auxiliary AI software, is used appropriately within the health care sector.

### Limitation

This study is a cross-sectional analysis, and the findings suggest that ChatGPT 4.0 possesses a certain level of nursing professional knowledge. However, high-quality prospective randomized controlled trials are still required to validate the actual effectiveness of ChatGPT 4.0 in nursing education, learning, and clinical application. Besides, since the logic behind how AI processes questions is part of the company’s “black box,” we can only understand its logic in processing inputs in different languages by interacting with the AI software. Therefore, we infer that the differences in handling Chinese and English inputs are due to variations in the amount of training between languages.

### Conclusion

This cross-sectional study collected and analyzed 618 nursing-related MCQs, including NCLEX-RN practice questions and NNLE actual exam questions, to evaluate the performance of ChatGPT 4.0 in processing different language inputs. The study exclusively used ChatGPT 3.5 for Chinese-to-English and English-to-Chinese translations and found that ChatGPT 4.0 demonstrated a significantly higher accuracy rate than ChatGPT 3.5 and Google Bard, particularly in handling English input for NCLEX-RN Practice MCQs and Chinese input for NNLE exam MCQs. These findings suggest that ChatGPT 4.0 has substantial potential as an effective learning assistance tool for nursing education and can provide valuable information and reference in clinical nursing settings due to its advanced real-time text generation capabilities.

## Supplementary material

10.2196/52746 Multimedia Appendix 1 File for the original dataset.

10.2196/52746 Multimedia Appendix 2 STROBE checklist cross-sectional.

## Abbreviations

AI: artificial intelligence
LLM: large language model
MCQ: multiple-choice question
NCLEX-RN: National Council Licensure Examination for Registered Nurses
NNLE: National Nursing Licensure Examination
