# Correction: Performance of ChatGPT on Nursing Licensure Examinations in the United States and China: Cross-Sectional Study

**DOI:** 10.2196/105550

**Published:** 2026-07-24

**Authors:** Zelin Wu, Wenyi Gan, Zhaowen Xue, Zhengxin Ni, Xiaofei Zheng, Yiyi Zhang

**Affiliations:** 1 Department of Bone and Joint Surgery and Sports Medicine Center, The First Affiliated Hospital of Jinan University, No.613, Huangpu Avenue West, Tianhe District, Guangzhou, Guangdong, 510630, China, 86 18002255355; 2 Department of Joint Surgery and Sports Medicine, Zhuhai People's Hospital (Zhuhai Clinical Medical College of Jinan University), Zhuhai, Guangdong, China; 3 School of Nursing, Yangzhou University, Yangzhou, Jiangsu, China

In “Performance of ChatGPT on Nursing Licensure Examinations in the United States and China: Cross-Sectional Study” [1], an error was identified.

The affiliation of authors ZW, ZX, XZ, and YZ was incorrectly listed as:

 Department of Bone and Joint Surgery and Sports Medicine Center, The First Affiliated Hospital, Guangzhou, China

This has been corrected to the following:

 Department of Bone and Joint Surgery and Sports Medicine Center, The First Affiliated Hospital of Jinan University, Guangzhou, China

This correction appeared in the online version of the article on the JMIR Publications website, together with this correction notice. Because this correction was made after submission to PubMed, PubMed Central, and other full-text repositories, the corrected article was resubmitted to these repositories.
